# Process of Pure Copper Fabricated by Selective Laser Melting (SLM) Technology under Moderate Laser Power with Re-Melting Strategy

**DOI:** 10.3390/ma16072642

**Published:** 2023-03-27

**Authors:** Rong Hu, Kangjing Su, Zibin Lao, Yixun Cai, Bin Fu, Matthew M. F. Yuen, Zhaoli Gao, Mingxuan Cao, Ying Wang

**Affiliations:** 1 School of Applied Physics and Materials, Wuyi University, Jiangmen 529020, China; 2 Department of Intelligent Manufacturing, Wuyi University, Jiangmen 529020, China; 3 School of Innovation and Entrepreneurship, Wuyi University, Jiangmen 529020, China; 4 Department of Mechanical Engineering, Hong Kong University of Science and Technology, Hong Kong 999077, China; 5 Biomedical Engineering Department, The Chinese University of Hong Kong, New Territories, Hong Kong 999077, China

**Keywords:** pure copper, selective laser melting, re-melting strategy, relative density, surface roughness

## Abstract

Pure copper (Cu) material, because of its high thermal conductivity, can be 3D printed to fabricate effective thermal management components. However, in the selective laser melting (SLM) process, due to copper’s high optical reflectivity, Cu-based parts need to be printed using high laser power. In this study, we demonstrated 3D printing with a re-melting strategy is able to fabricate high-density and low-surface-roughness pure copper parts using only a moderate laser (350 W) power. The effect of the re-scan to initial scan speed ratio on the printing quality resulting from the re-melting strategy is discussed. The re-melting strategy is likened to a localized annealing process that promotes the recrystallization of the newly formed copper microstructures on the re-scan path. Given a hatch spacing of 0.06 mm and a powder layer thickness of 0.05 mm, Cu samples with 93.8% density and low surface roughness (Sa~22.9 μm) were produced using an optimized scan speed of 200 mm/s and a re-scanning speed of 400 mm/s, with a laser power of 350 W. Our work provides an approach to optimize the laser power for printing pure copper 3D parts with high relative density (low porosity) and low surface roughness while ensuring the lifetime stability of the part. The re-melting strategies have broad implications in 3D printing and are particularly relevant for metals with high reflectivity, such as pure copper.

## 1. Introduction

The ability of additive manufacturing (AM) to fabricate complex metal parts by aggregating metal powder in an incremental, layer-by-layer fashion, guided by a computer-aided design (CAD) model, makes it an attractive technology [1,2,3,4]. Metal 3D printing using selective laser melting (SLM) employs a high-power laser to selectively melt and fuse metal powder layers to create three-dimensional objects [5,6]. Due to its additive nature, 3D components with complicated shapes and intricate surfaces or volumes can be created with little material wastage [7,8].

Copper is a metallic metal that has exceptional electrical (58 × 10^6^ S/m, or 100% International Annealed Copper Standard (IACS)) and thermal conductivity (400 W/(mK)) properties [9,10]. As a result, it is frequently utilized in applications such as heat sinks and exchangers where these parameters are crucial [11]. Such Cu-based parts can be designed with fine features to increase their surface area and heat transfer efficiency [12]. However, pure copper has poor optical absorptivity (high reflectivity) in the near-IR wavelength and high thermal diffusivity, which makes it difficult to retain the input laser energy for localized melting. This creates challenges for fabricating fully dense copper parts in an SLM system with moderate laser power (200–400 W) [13]. For the majority of laser wavelengths, the reflection ratio of the incident laser beam by pure Cu is often higher than 90%, resulting in less than 10% of the beam energy being absorbed during printing. This prevents densification, hinders fusion, and leads to low relative density [14]. The high laser reflectivity may result in scattered or reflected beam rays inside the build chamber, which could seriously harm the 3D printer’s optical system [15]. The relatively high thermal conductivity of copper deters the formation of an ideal molten pool because heat from laser irradiation can rapidly diffuse to the neighboring region [16].

In recent years, researchers have attempted to overcome the above-mentioned basic deficiencies of pure copper SLM using the following approaches:

(1) One approach is using shorter wavelength lasers (e.g., blue laser at 488 nm) to improve the absorption of pure copper [17]. However, high-power blue lasers are more expensive, and more importantly, the 488 nm laser source is a semiconductor laser with relatively poor beam quality, resulting in a much lower printing accuracy.
(2) Another approach is using a high energy input and high laser power to improve printing quality. Most of the work used lasers with power between 600 W and 1 kW to attain a maximum relative density of 99.4% [18,19]. However the high amount of backscattered and reflected light during processing may harm the laser modules [15].
(3) A third approach is adding a light-absorbing coating, such as carbon materials or alloys, on the Cu powder to improve absorption [20,21]. However, these additives can lead to a reduction in the thermal conductivity of the material.

The above approaches suffer from their own inherent shortcomings. The intricate thermal history created by the continuous laser heating and cooling procedure during the SLM of the Cu influences not only the microstructure but also the mechanical performance. Laser re-scanning, a technique where the solidified layer is once more melted and hardened by further laser scanning before the deposition of the next layer, is a promising method for generating fast cemented microstructure. By using the same process settings as the initial scan, Vaithilingam et al. [22] found that the surface roughness of the top surface was reduced. According to Kruth et al. [23], on the effect of the re-scan speed on residual stress, the residual stress after the re-scan decreases only when the re-scan speed is low enough to re-melt the material. If the pace of the re-scan is too fast, the residual stress even grows after the re-scan. However, Mercelis et al. [24] noted that if 50% of the forming energy is utilized during re-scanning, a 30% reduction in residual stresses can be achieved. If 100% of the forming energy is utilized, no reduction in residual stresses is achieved. After simulating the SLM forming process of Ti6Al4V with ANSYS, Miao et al. [25] proposed a re-scanning technique to reduce the residual stresses. The re-scanning process parameters were optimized to reduce the average residual stress from 322 MPa to 254 MPa. Nowadays, re-melting strategies have been successfully applied to stainless steel [26], AlSi10Mg [27], titanium aluminides [28], and other materials [29,30] to increase part density [31], reduce surface roughness [32,33], release entrapped gases [34], and increase the wear resistance [35]. However, the influence of the re-melting strategy on SLM-manufactured pure copper has not been studied in detail. In order to satisfy the demands of reducing laser power and enhancing the sample’s density, the re-melting strategy could be a promising candidate for Cu 3D printing. This method could temporarily slow down the sample’s cooling rate, minimize the internal stress brought on by the sample’s fast temperature change, and eliminate defects such as porosities, infusions, and microscopic cracks.

In this study, we improved the printability of pure copper by laser re-melting without substrate preheating. By comparing the printing quality of pure copper using a single melting strategy (SMS) against the re-melting strategy (RMS), it has been demonstrated that pure copper samples of high density and low surface roughness can be produced with a modest laser power (350 W) using RMS. Following the process optimization, dense Cu components (93.8%) with low surface roughness (Sa ~22.9 μm) were generated at a scan speed of 200 mm/s (re-melting scanning speed 400 mm/s), a hatch spacing of 0.06 mm, and a layer thickness of 0.05 mm. The effect of the re-scan to initial scan speed ratio on the printing quality was also examined. The method developed in this study could also apply to other highly reflective materials such as pure gold, pure silver, etc.

## 2. Materials and Methods

### 2.1. Powder Characterization and SLM Equipment

**Figure 1:**
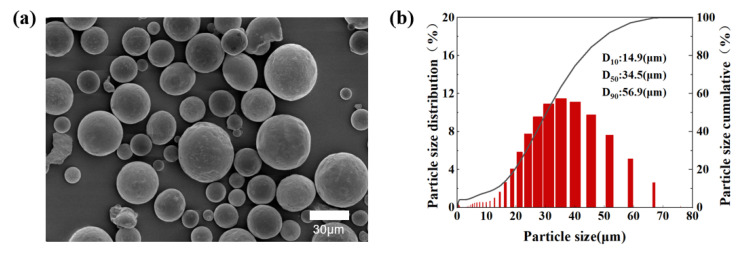
(**a**) Morphology of the pure copper powder detected using SEM. (**b**) Particle size distribution of the pure Cu powder.

Gas-atomized commercial Cu powders (Beijing Youxinglian Nonferrous Metal Co. Ltd., Beijing, China) with a minimum purity of 99.9 wt % were selected as the raw materials. The chemical composition of this powder is presented in Table 1. The micrograph of a pure copper powder is exhibited in Figure 1a. The particle size distribution (D_10_ = 14.9 μm, D_50_ = 34.5 μm, and D_90_ = 56.9 μm) displayed in Figure 1b was measured utilizing a Mastersizer 3000 laser diffraction particle analyzer (Malvem Instruments Ltd., UK). Additionally, the flowability of the pure Cu powder (16.5 s/50 g) was measured by a BT-200 Hall flowmeter (Bettersize Instruments Ltd., Dandong, China), and the tap density (4.96 g/cm^3^) was measured according to the tapping density test (BT-301, Bettersize Instruments Ltd., China).

The relative density ($\rho_{RD}$) of the SLM Cu samples was measured by an analytical balance (ABZ 200C, PCE instruments, Meschede, Germany) based on Archimedes’ principle using the following Equation (1):

(1) $$\rho_{RD}=\frac{\rho_{SLM}}{\rho_{STANDARD}}=\frac{\rho_{\mathrm{water}}\cdot m_{SLM\left(\mathrm{air}\right)}}{\rho_{STANDARD}\cdot \left(m_{SLM\left(\mathrm{air}\right)}-m_{SLM\left(\mathrm{water}\right)}\right)}$$

where $\rho_{SLM}$ is the density of the SLM copper samples, $\rho_{STANDARD}$ is the standard density of copper, $m_{SLM\left(\mathrm{air}\right)}$ is the weight of the SLM samples in air, $m_{SLM\left(\mathrm{water}\right)}$ is the weight of the SLM samples in water, and $\rho_{\mathrm{water}}$ is the density of water. 

The weight of the SLM parts was measured using an analytical balance (ABZ 200C, PCE instruments, Germany), and the measurement was replicated three times to determine the mean value of the weight. Three samples were tested and averaged to determine the relative density. All the samples for metallographic analysis were first polished using SiC grinding papers followed by Al_2_O_3_ suspensions. The polished samples were etched by a reagent of 2 mL HCl, 1 mL Fe_3_Cl, and 97 mL C_2_H_5_OH for 50 s. The cross-section microstructures were then displayed via the optical microscope (Leica Dmi5000m, Germany). The phase analysis of the SLM Cu parts was performed by X-ray diffraction (XRD, Siemens, Munich, Germany) equipment.

A DiMetel-300 LPBF machine (Laseradd Technology Co Ltd., Guangzhou, China) was used to process the copper, which was equipped with a 500 W continuous wavelength (CW) fiber laser with a wavelength of 1070 nm (±10 nm) and a spot size of 75 μm (±5 μm). A vacuum and argon purge was used in the build chamber in order to keep the oxygen content below a maximum of 500 ppm.

### 2.2. Design of SLM Process with SMS

In order to better discuss the effect of the re-melting strategy on the sample molding quality, we first analyzed the influence of volumetric laser energy density (in terms of the laser power, laser scanning speed, and hatch spacing) and layer thickness on the single melting process (Table 2). A stripe laser scan strategy was used for all samples. The structures designed with SOLIDWORKS were uploaded to the Materialise Magics 23.0 for parameter assignment. The parts were then printed on a copper building plate (copper, 250 × 250 × 25 mm^3^). The volumetric laser energy density $E_{v}$ (J/mm^3^) was calculated with the following Equation (2):

(2) $$E_{v}=\frac{P}{V\cdot H\cdot T}$$

where P is the laser power (W), T is the layer thickness (mm), H is the hatch spacing (mm), and V is the laser scan speed (mm/s).

### 2.3. Design of SLM Process with RMS

**Figure 2:**
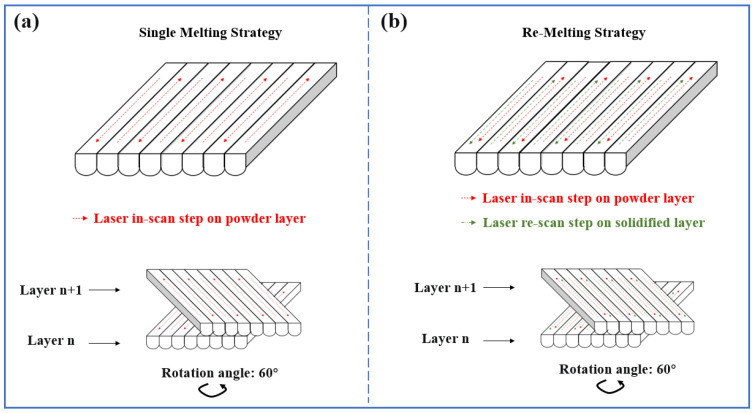
Schematic diagram of laser scanning strategies (**a**) SMS and (**b**) RMS.

The RMS was introduced in the SLM of Cu. Each layer was subjected to two consecutive heating steps: the initial scan and the re-scan, as shown in Figure 2. Thirty cubes of 10 × 10 × 10 mm^3^ were produced. The total number of build layers was 200, and the dwell time between layers was 5 s. The process parameters of the SLM process with RMS are displayed in Table 3, where the scanning speed of the re-scan step was set to one to five times that of the initial scan step (the data in the initial scan, including the scanning speed, hatch spacing, and layer thickness, are explained in Section 3.2). The scanning direction between successive layers was rotated by 60° layer by layer.

## 3. Result and Discussion

### 3.1. Phase Transformation

**Figure 3:**
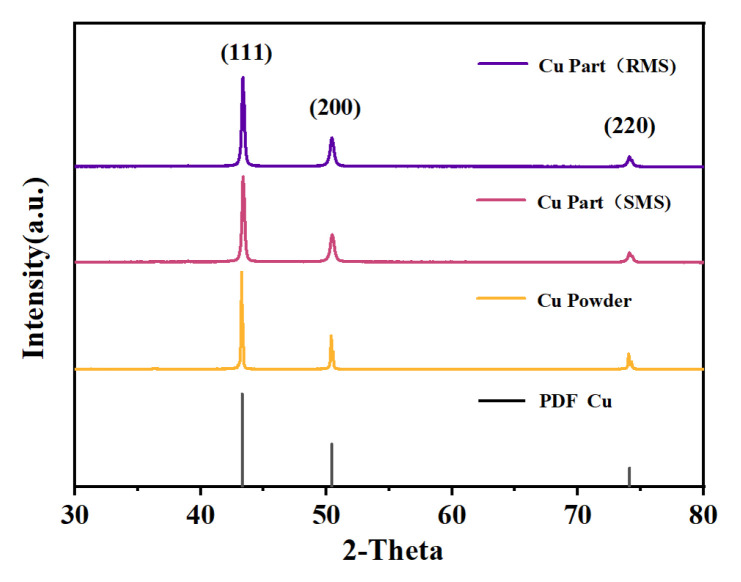
XRD patterns of copper powder and copper parts with SMS or RMS (laser power 350 W, initial scanning speed 200 mm/s, re-scanning speed 400 mm/s, hatch spacing 0.06 mm, and layer thickness 0.05 mm).

Figure 3 shows the typical XRD patterns of the Cu powder and the SLM samples. The diffraction peaks of the copper powder before and after laser melting did not change, and only peaks associated with the pure Cu phase were detected. The diffraction peaks at 2θ = 43.3, 50.4, and 74.1° were attributed to the (111), (200), and (220) planes of the face-centered Cu [36]. In addition, it can be further concluded that the SLM Cu samples showed no significant oxidation during the fabrication process.

### 3.2. The SLM Process with SMS

**Figure 4:**
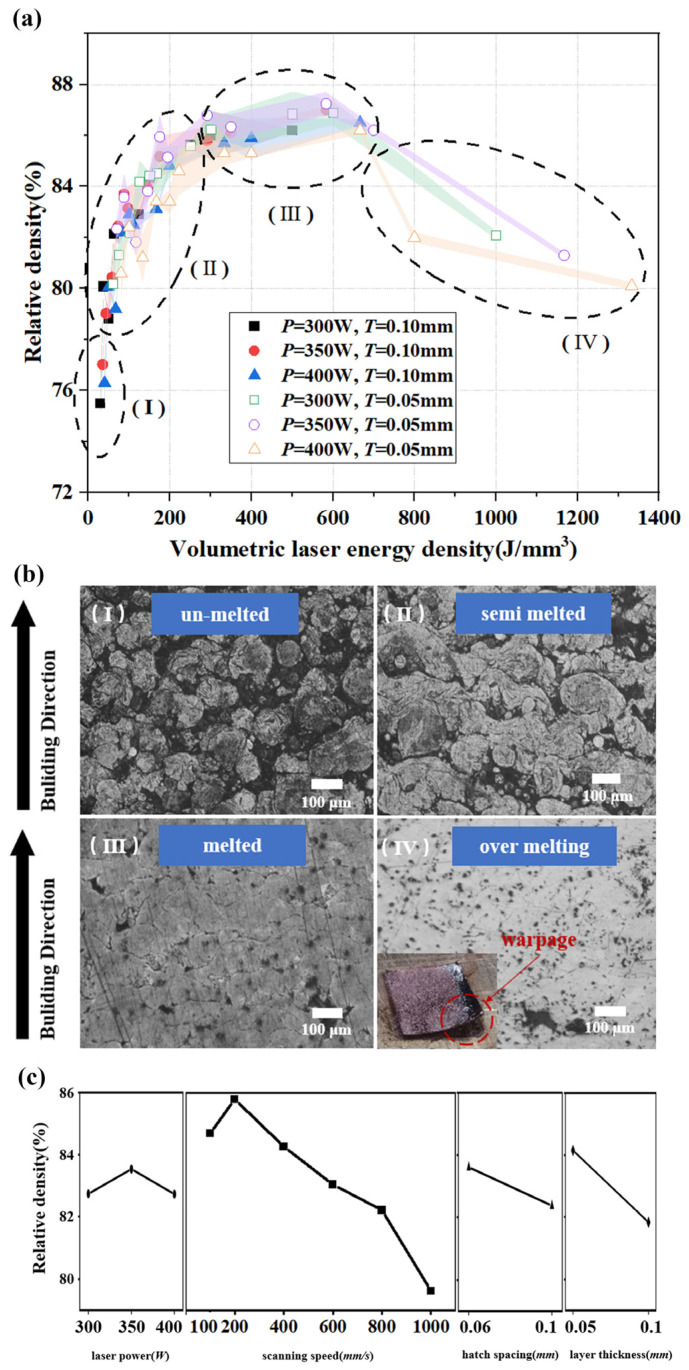
(**a**) Relative density of the fabricated copper samples with different process parameters in the function of the volumetric laser energy density. (**b**) Cross-sectional surfaces of metal samples observed under an optical microscope (I–III) and pictures of over-melting samples (IV). (**c**) Trends of factors and relative density.

The SLM process optimization was performed by employing a scan speed of 100–1000 mm/s and laser power of 300–400 W, layer thicknesses of 0.05 mm and 0.1 mm, and hatch spacings of 0.06 mm and 0.1 mm. Subsequently, a graph of the applied volumetric laser energy density versus the relative density of the parts was plotted, as shown in Figure 4a.

In Figure 4a, a clear trend of variation in the component density can be seen, when the volumetric laser energy density gradually increased, while un-melted (I), semi-melted (II), melted (III), and over-melted (IV) cubes (10 × 10 × 10 mm^3^) were formed. We divided the laser power density into four regions according to the different forming effects. As seen in region I, the sample densities were less than 77%. Due to insufficient energy supply, a large amount of un-melted powder remained within the melt pool that was not fully developed, resulting in samples with weakly adhered particles as seen in the cross-section microscopic images in Figure 4b(I). Region II shows that as the volumetric laser energy density increased, the relative density climbed rapidly. As shown in the cross-sectional micrograph of the sample in Figure 4b(II), the powder in each layer melted more fully because of the boost in energy input. However, the effective energy input in this region was still insufficient, resulting in poor binding between the layers. Delamination and un-melted powder were observed at the layer boundaries. As seen in region III, the relative density of the samples tended to vary steadily (300 to 700 J/mm^3^). In Figure 4b(III), the cross-section micrograph shows the absence of un-melted powder, reflecting a fully developed melt pool. However, many pores and cracks were present. Combining the effects of the factors on the relative densities, as shown in Figure 4c, when the volumetric laser energy density reached 583 J/mm^3^ (*P* = 350 W, *V* = 200 mm/s, *H* = 0.06 mm, *T* = 0.05 mm), the sample density reached its maximum ($\rho_{RD}$= 87.6 ± 0.21%) under the SMS. Once the volumetric laser energy density exceeded 700 J/mm^3^ (region IV), the localized energy of the powder bed was too large, thus causing powder spattering, so it increased the porosity. Over-melting occurred at the edge of the sample during the printing process, and excessive internal stress caused severe warping of the sample, as shown in Figure 4b(IV). In addition, with a layer thickness of 0.10 mm, the power density during printing proved challenging to achieve the ideal situation. As the layer thickness increased, spatters and vapored metal were more likely to occur and induced large porosity with discontinuous laser trajectories on the sample surface [36]. Therefore, the layer thickness of 0.05 mm was employed in the subsequent experiments.

### 3.3. The SLM Process with RMS

**Figure 5:**
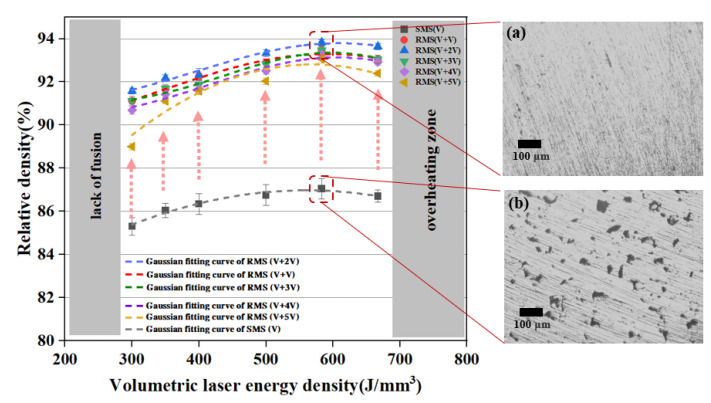
Relationship between the volumetric laser energy density and corresponding relative density for Cu parts (*V* = 200 mm/s). The insets (**a**,**b**) are the corresponding micrographs of the polished sample for the RMS and SMS processing strategies.

The relative densities of the similar pure copper cubes prepared by SLM with single melting and RMS are shown in Figure 5. Each sample’s relative density was tested three times. It was demonstrated that dense samples (above 85%) can be produced by both strategies with volumetric laser energy density in the 300–667 J/mm^3^ range. However, the RMS sample relative density was significantly higher than the corresponding SMS sample produced with the same volumetric laser energy density. A layer thickness of 0.05 mm was used in the RMS sample fabrication as a shallower layer thickness would facilitate better penetration of laser power to re-melt the solidified initial scan material. A micrograph of the cross-section of a typical sample prepared by each of the two processing strategies is shown as insets (a) and (b) in Figure 5. The defects of the RMS sample are significantly less than those in the SMS sample. This shows that re-melting allowed for better fusion of the powder, both within the layer and across the layers resulting in lower porosity. As explained by Kenel et al. [37], re-scanning enabled the deposit of higher total radiant exposure, which slowed down the cooling rate of the samples and lowered the internal stress, leading to a reduction of pores, cracks, and other defects. Moreover, the re-scanning allows the re-melting of the material in the original scan path to facilitate recrystallization of the material and redistribution of the stress. The forming quality of samples was also significantly influenced by the volumetric laser energy density. It should be noticed that the low energy density (<300 J/mm^3^) resulted in a reduction in the relative density of the samples, due to insufficient melting and the formation of lack-of-fusion pores, as suggested by Jadhav et al. [38]. As shown in Figure 5, the relative density increased with volumetric laser energy density from 300 onward to a maximum of 93.4% at 587 J/mm^3^. This corresponded to 350 W, which is a relatively moderate laser power for the SLM process. The result in Figure 5 also shows the relative density value for RMS with different re-scan speeds. The annotation V + V represents the case when the re-scan speed is the same as the scan speed, while V + xV represents the case when the re-scan speed is x times the scan speed. The results indicate that the V + 2V case produces samples with the highest relative density. The effect of the re-scan speed is therefore a parameter that warrants further investigation.

**Figure 6:**
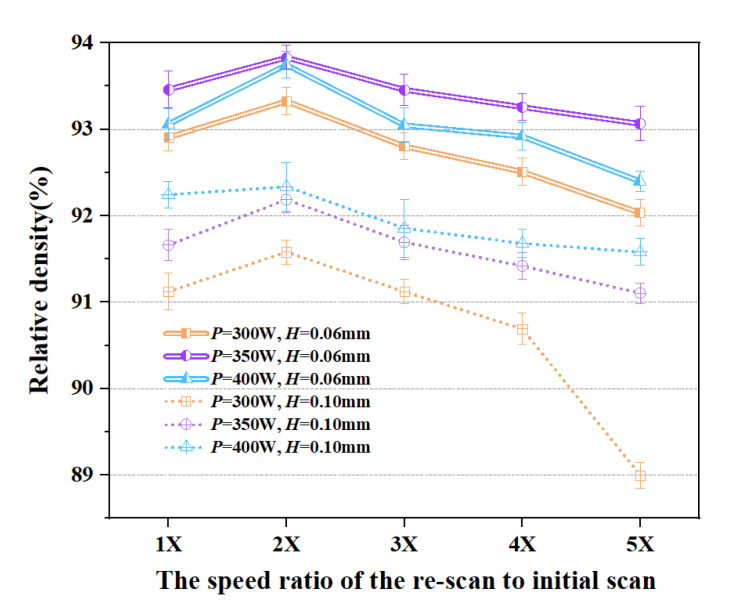
The relationship between the re-scan to initial scan speed ratio and the relative density (the initial scan speed was fixed at 200 mm/s).

**Figure 7:**
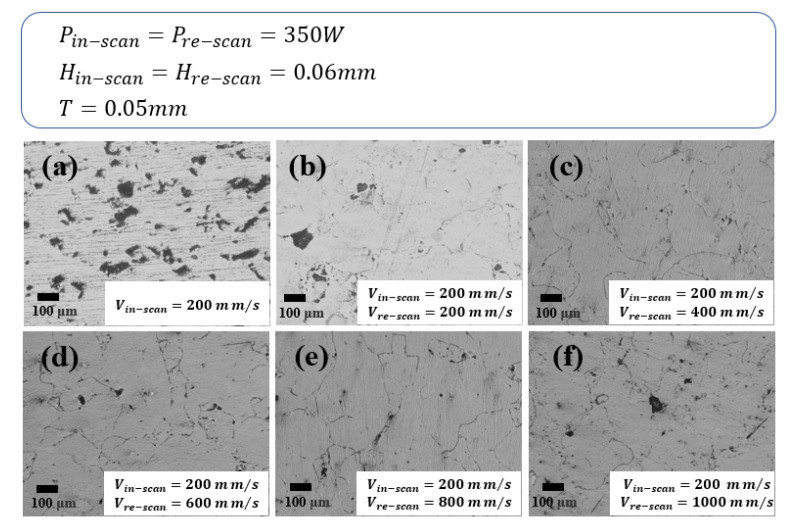
Micrographs of polished surfaces under SMS (**a**) and RMS (**b**–**f**).

Figure 6 shows the relationship between the re-scan to initial scan speed ratio and the relative density of the samples. As explained in Equation (2), increasing the laser scanning speed results in a decrease in the volumetric laser energy density. When the re-scanning speed equals that of the initial scan, the energy input into the sample is the same as the initial scan leading to a repetition of the earlier melting and solidification process of the SMS crystalline structure. This would likely fully melt the solidified SMS crystalline structure, resulting in the destruction of the microstructure already formed in the initial scanning step [39]. Increasing the re-scan speed reduced the energy input and thus reduced the temperature of the solidified SMS crystalline structure along the re-scan path. In fact, depending on the re-scan speed, the temperature may not even reach the melting temperature of copper to form a melt pool along the re-scan path. If the energy input is sufficient to enable recrystallization of the microstructure along the re-scan path, the process would be likened to the annealing of the microstructure, except that this is a localized annealing process with a short recrystallization time. If the re-scan speed is too fast, the re-scan energy input would be insufficient to facilitate the recrystallization process. Therefore, it is expected that there is an optimized re-scan speed for localized annealing that neither overly re-melts nor under-energizes the process. This is analogous to the determination of the optimized annealing temperature of copper for the given SLM process condition. For the current layer thickness and hatch spacing, the maximum relative density ($\rho_{RD}$ = 93.8%) was obtained for Cu samples at a re-scanning speed of twice the initial speed in the range of re-scan speeds tested. The micrograph of the samples also demonstrates the above results. The porosity of samples observed on the surfaces in Figure 7 shows that minimum porosity occurs when the re-scan rate is twice the initial scanning rate.

### 3.4. Roughness Analysis

**Figure 8:**
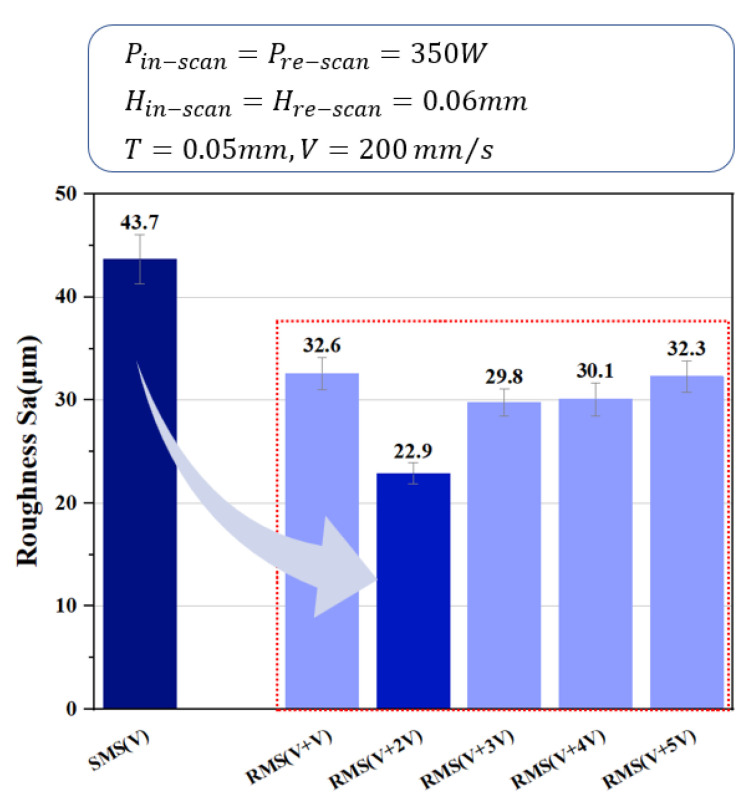
Influence of re-scan speed on surface roughness of the sample.

In the SLM process, copper’s high thermal conductivity quickens the melt pool’s heat dissipation and shortens the solidification process, yet the melt pool takes a relatively long time to form in the process, thus causing balling to occur in the current sample. Additionally, rapid heat dissipation from the melt pool generates a high temperature gradient, inducing the formation of cracks. The effects of the SMS and RMS strategies on the top surface roughness of the samples are shown in Figure 8. The RMS resulted in a reduced surface roughness of all samples compared with that of SMS. However, the improvement of the surface roughness was best when the re-scanning speed was two times higher than the scanning speed. When the speed of the sample re-scanning was lowered, the volumetric laser power density re-melted the solidified microstructure under the re-scan path with higher energy input and thus repeated the previous crystallization process, resulting in a minor change in the surface roughness of the sample. When the re-scanning speed was too fast, the volumetric laser power density was inadequate to facilitate effective annealing and recrystallization of the microstructure. However, with an appropriate re-scanning speed to facilitate localized annealing, this allows for stress redistribution and porosity reduction, which leads to an improvement in the surface roughness. This suggests that the re-melting strategy could be optimized to cater to a favorable condition for the localized annealing of the microstructure under the re-scan path with a prescribed process condition.

## 4. Conclusions

In this work, the additive manufacturing of pure copper samples, with high relative density and low surface roughness, was effectively accomplished using the SLM re-melting strategy (RMS) with medium laser power (350 W). The critical parameter in this work is the volumetric laser energy density that governs the RMS process. It must be highlighted that the re-melting strategy is also affected by the powder layer thickness and the hatch spacing as they are the parameters that jointly define the volumetric laser energy density. Too thick a layer thickness will hamper the laser energy having an effect on the inter-layer re-melting and recrystallization. Too large hatch spacing will not facilitate the aggregation of microstructures across the scan path. These are parameters that can be further optimized in future work. The re-melting strategy is likened to a localized annealing process, effectively redistributes the thermal stress, reduces the formation of defects, and facilitates the formation of a more homogeneous microstructure inside the sample. This has the advantage over subsequent conventional annealing of 3D metal printing parts as the recrystallization can be conducted within the printing process, rather than having a follow-up process. This strategy has a further advantage that it offers more flexibility to handle localized microstructure issues for parts with intricate geometry as the process takes a localized rather than bulk annealing approach. The impact of the re-scan and initial scan speed ratio on the quality of the printing was explored in this work. It has been demonstrated that an optimized speed ratio exists for the RMS process. After process optimization, dense Cu components (93.8%) with low surface roughness (Sa ~22.9 μm) were generated at a scan speed of 200 mm/s (the re-scanning speed being 400 mm/s), a hatch spacing of 0.06 mm, and a layer thickness of 0.05 mm with a moderate laser power of 350 W. The current work can be extended by adopting machine learning and simulation of the recrystallization process under the re-melting strategy to further optimize the parameters for fabricating quality metal parts with a high relative density, low porosity, low surface roughness, good surface finish, and more homogeneous microstructure using moderate laser power. This approach can thoroughly exploit the advantages of 3D printing to make a big impact in thermal management, especially in the design of products for microfluidic heat sinking. Its application could be further explored in the 3D printing of highly reflective metal items in the jewelry industry.

## Figures and Tables

**Table 1:** Chemical composition of pure Cu powder employed in this research.

Elements	Fe	Zn	Pb	As	Cd	P	S	TAO	Cu
Weight (wt.) %	0.018	0.036	0.010	0.005	0.005	0.075	0.050	0.150	Balance

**Table 2:** SLM parameters and the corresponding value or range to be tested.

Parameter	Value or Range
**Laser power (W)**	300, 350, 400
**Laser scan speed (mm/s)**	100, 200, 400, 600, 800, 1000
**Layer thickness (mm)**	0.05, 0.10
**Hatch spacing (mm)**	0.06, 0.10

**Table 3:** Process conditions for the RMS.

Boundary Conditions	Substrate: Copper Build Plate with a Thickness of 25 mm Scanning Strategy: X-Y Sequential Layers Scanning with 60° Orientation			
Process Parameter (unit)	Laser Power P (W)	Scanning Speed V (mm/s)	Hatch Spacing H (mm)	Layer Thickness T (mm)
Initial scan	300, 350, 400	200	0.06, 0.10	0.05
Re-scan	Same data as above	One to five times the initial scan speed	Same data as above	0.05

## Data Availability

Not applicable.
